# In silico feasibility of novel biodegradation pathways for 1,2,4-trichlorobenzene

**DOI:** 10.1186/1752-0509-4-7

**Published:** 2010-02-02

**Authors:** Stacey D Finley, Linda J Broadbelt, Vassily Hatzimanikatis

**Affiliations:** 1 Department of Chemical and Biological Engineering, McCormick School of Engineering and Applied Sciences, Northwestern University, 2145 Sheridan Road, Evanston, IL 60208, USA; 2 Laboratory of Computational Systems Biotechnology, Ecole Polytechnique Fédérale de Lausanne (EPFL) and Swiss Institute of Bioinformatics (SIB), CH H4 625, Station 6, CH-1015 Lausanne, Switzerland

## Abstract

**Background:**

Bioremediation offers a promising pollution treatment method in the reduction and elimination of man-made compounds in the environment. Computational tools to predict novel biodegradation pathways for pollutants allow one to explore the capabilities of microorganisms in cleaning up the environment. However, given the wealth of novel pathways obtained using these prediction methods, it is necessary to evaluate their relative feasibility, particularly within the context of the cellular environment.

**Results:**

We have utilized a computational framework called BNICE to generate novel biodegradation routes for 1,2,4-trichlorobenzene (1,2,4-TCB) and incorporated the pathways into a metabolic model for *Pseudomonas putida*. We studied the cellular feasibility of the pathways by applying metabolic flux analysis (MFA) and thermodynamic constraints. We found that the novel pathways generated by BNICE enabled the cell to produce more biomass than the known pathway. Evaluation of the flux distribution profiles revealed that several properties influenced biomass production: 1) reducing power required, 2) reactions required to generate biomass precursors, 3) oxygen utilization, and 4) thermodynamic topology of the pathway. Based on pathway analysis, MFA, and thermodynamic properties, we identified several promising pathways that can be engineered into a host organism to accomplish bioremediation.

**Conclusions:**

This work was aimed at understanding how novel biodegradation pathways influence the existing metabolism of a host organism. We have identified attractive targets for metabolic engineers interested in constructing a microorganism that can be used for bioremediation. Through this work, computational tools are shown to be useful in the design and evaluation of novel xenobiotic biodegradation pathways, identifying cellularly feasible degradation routes.

## Background

The prevalence and widespread use of man-made chemicals ("xenobiotics") has led to a focused effort to establish new technologies to reduce or eliminate these contaminants from the environment. Commonly used pollution treatment methods such as incineration, landfilling, and air stripping also have an adverse effect on the environment [1,2]. Additionally, these methods are costly and sometimes inefficient. Therefore, it is important to develop alternative methods of biodegradation that are effective, minimally hazardous, and economical. One promising treatment method is to exploit the ability of microorganisms to use these foreign substances for maintenance and growth, a process known as bioremediation [3].

Microorganisms provide a wealth of potential in biodegradation. It has been proposed that the ability of these organisms to reduce the concentration of xenobiotics is closely linked to their long-term adaptation to environments where these compounds exist [4-6]. Genetic engineering may be used to enhance the performance of the microorganisms such that they have the desired properties needed for biodegradation. Genetically engineered microorganisms (GEMs) have new metabolic pathways, more stable catabolic activity, and expanded substrate ranges relative to existing organisms [7]. For example, genetic engineering has been employed to design specific pathways [8] or a microbial consortium [9] for the biodegradation of an organophosphorus insecticide. Whole-genome sequencing has also proved helpful in understanding and enhancing microorganisms for bioremediation [10].

In order to fully explore the capabilities of microorganisms in cleaning up the environment, the use of computational tools to predict novel biodegradation pathways for pollutants and gain a better understanding of the fate of these compounds in the environment would be valuable [11]. Prediction methods such as the Pathway Prediction System (PPS) [12], META [13], and others [14-18] rely on databases of rules describing biotransformations that occur in cellular and environmental processes. An alternative method is the Biochemical Network Integrated Computational Explorer (BNICE), a framework developed for the discovery of novel biochemical reactions [19-21]. BNICE has been shown to be a pathway prediction method that generates feasible biodegradation routes [22]. BNICE utilizes reaction rules derived from the Enzyme Commission (EC) classification system, which provide a compact way to describe biochemical reactions and can be used to link the degradation of xenobiotic compounds to small molecule metabolism.

Given the wealth of novel biodegradation pathways obtained using computational prediction methods, it is necessary to evaluate their relative feasibility. Thermodynamic feasibility is a useful metric to evaluate potential biodegradation pathways. In the absence of experimental data for the Gibbs free energies of formation and reaction, group contribution provides an estimate of the thermodynamic properties of compounds and reactions [23] and is an effective tool in the evaluation [24,25] and reconstruction [26,27] of genome-scale models. Additionally, metabolic flux analysis (MFA) provides a means of investigating the cellular feasibility of novel pathways; that is, how implementation of the pathway influences the existing metabolism of an organism and gives rise to competition for cellular resources. MFA can be augmented with thermodynamic constraints, a methodology called thermodynamics-based metabolic flux analysis (TMFA) [24], in order to generate thermodynamically feasible flux profiles and predict cellular behavior. These tools provide a systematic evaluation of the feasibility of novel pathways within the context of the cellular environment.

In this work, we describe the evaluation of novel pathways to degrade 1,2,4-trichlorobenzene (1,2,4-TCB) in the context of the cellular metabolism of *Pseudomonas putida*, a pollutant-degrading organism. 1,2,4-TCB is one of the most widely used chlorobenzenes [28] and has many industrial uses. Chlorobenzenes have toxic effects in humans and animals [29,30], and 1,2,4-TCB in particular is included on the list of Priority Chemicals, as designated by the Environmental Protection Agency (EPA) http://www.epa.gov/epawaste/hazard/wastemin/priority.htm. A biodegradation pathway for 1,2,4-TCB has been proposed and is catalogued in the University of Minnesota Biocatalysis/Biodegradation Database (UM-BBD) [31]. This pathway is based on experimental results from various *Pseudomonas *species [32-34], where 1,2,4-TCB is metabolized to form chloroacetate and succinate. In addition, chloroacetate can be further metabolized to yield glycolate [35]. Here, we have used a model of *P. putida *KT2440 metabolism [36] to study the feasibility of the novel pathways obtained from BNICE. This particular strain is the only non-pathogenic *Pseudomonas *species for which a metabolic model has been created [36-38]. We augmented the model with novel biodegradation pathways obtained from BNICE, and MFA was used to predict the maximum biomass production using 1,2,4-TCB as the sole carbon source. The reactions in the augmented model were classified to determine the subset of reactions required for maximum production of biomass. We identified key characteristics of the flux profiles and nutrient utilization leading to differing amounts of biomass production. Additionally, this analysis revealed an inverse relationship between the production of biomass and metabolism of 1,2,4-TCB. Lastly, TMFA was performed to predict flux profiles free of thermodynamic infeasibilities. Ultimately, we identified novel pathways that were attractive alternatives to the known route, demonstrating the applicability of computational tools to evaluate the cellular feasibility of novel biodegradation pathways.

## Results

### Generation of novel biodegradation pathways

BNICE generated a reaction network involving a wealth of novel pathways describing the biodegradation of 1,2,4-TCB to form compounds with known metabolism. The enzyme actions involved in the known pathway were encoded in the BNICE framework and applied to 1,2,4-TCB for 10 generations. In total BNICE generated 1,031 compounds and 3,760 reactions [22]. Using this network of reactions, we searched for pathways from 1,2,4-TCB to compounds whose intermediary metabolism is well known and catalogued in the KEGG database [39], up to a pathway length of 15 reaction steps. We then screened the pathways to only study those whose products had known metabolism and were native to *P. putida*, and were shorter than or equal to the length of the known pathway. As a result of this screening process, we identified 27,860 novel pathways. The overall reaction of a pathway, i.e., the pathway stoichiometry, was used to classify the pathways (Table 1) and provided a compact way to compare the products and cofactors involved. Overall reaction [K] corresponds to the known pathway; that is, the pathway whose reactions have been shown to occur in various *Pseudomonas *species [32-35,40].

**Table 1:** Distinct overall reaction of novel biodegradation pathways

Abbreviation	Reaction equation^a^
[K]^b^	1,2,4-TCB + (2) O_2 _+ NADH + (3) H_2_O + (2) e^- ^→ NAD+ + (3) Cl^- ^+ (3) H^+ ^+ Glycolate + Succinate
[1]	1,2,4- TCB + (2) O_2 _+ (3) H_2_O + (2) e^- ^→ (4) H^+ ^+ (3) Cl^- ^+ Fumarate + Glycolate
[2]	1,2,4- TCB + (2) O_2 _+ (4) H_2_O + NADH → NAD^+ ^(5) H+ + (3) Cl^- ^+ Glycolate + (S)-Malate
[3]	1,2,4- TCB + (2) H_2_O + (2) e^- ^→ (3) Cl^- ^+ H^+ ^+ Catechol
[4]	1,2,4- TCB + (2) O_2 _+ (3) H^+ ^+ (6) e^- ^→ (3) Cl^- ^+ Catechol
[5]	1,2,4- TCB + (2) O_2 _+ NADH + H^+ ^+ (4) e^- ^→ NAD^+ ^+ (3) Cl^- ^+ Muconolactone
[6]	1,2,4- TCB + O_2 _+ (2) H_2_O + NADH → NAD^+ ^+ (3) Cl^- ^+ (3) H^+ ^+ Muconolactone
[7]	1,2,4- TCB + (2) O_2 _+ H_2_O + (4) e^- ^→ (3) Cl^- ^+ H^+ ^+ 2-Hydroxymuconate

^a ^We identified novel pathways whose non-cofactor products were compounds with known intermediary metabolism and native to *P. putida *metabolism.

^b ^The overall reaction corresponding to the known pathway.

### Growth on 1,2,4-TCB

**Figure 1:**
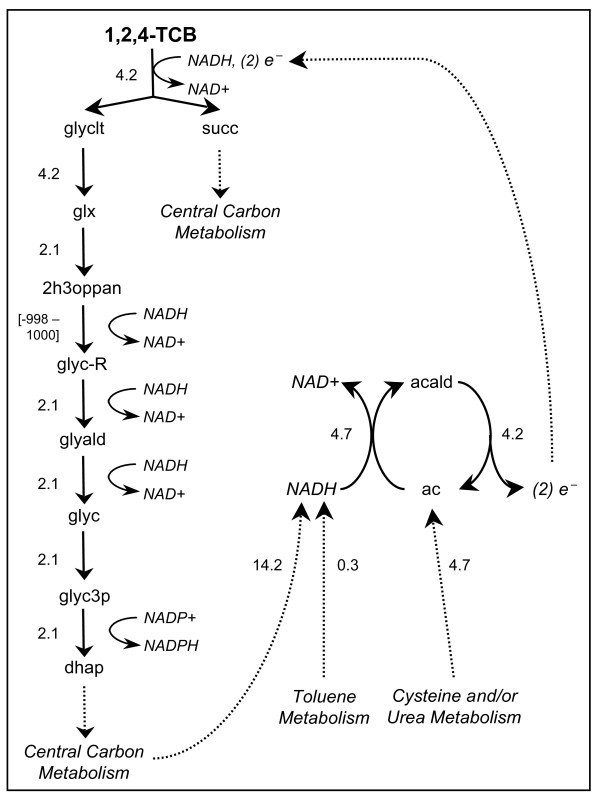
***P. putida *reactions for growth on 1,2,4-TCB**. The reaction network used to integrate the degradation products of 1,2,4-TCB into *P. putida *metabolism and the cellular processes involved in generating the required reducing power (electrons and/or NADH) are shown. Central carbon metabolism includes the Entner-Doudoroff pathway, gluconeogenesis, and the TCA cycle. The acetaldehyde dehydrogenase (acetylating) reaction, involved in toluene metabolism, was also used to generate NADH. The overall reactions for eight different pathways were implemented individually; the reaction network resulting from implementation of overall reaction [K] is given here. Reaction networks for all eight overall reactions studied are shown in Additional File 1. The units for the flux values are mmol/gDW/h. Compound abbreviations are given in the appendix.

Each novel biodegradation reaction pathway allowed the organism to utilize 1,2,4-TCB as a carbon and energy source. We applied metabolic flux analysis to study the effect of engineering the novel biodegradation pathway into *P. putida *and to learn how the pathways influenced the existing metabolism of the cell. Figure 1 shows the route used to integrate the degradation products from overall reaction [K] into the metabolic network of *P. putida *(see Additional File 1 for the reaction network for all overall reactions examined). In each case, when optimizing biomass production, implementation of the novel reaction resulted in some amount of biomass (Table 2), indicating that 1,2,4-TCB metabolism was linked to cell growth. Overall reaction [3] was superior to the others, generating the largest amount of biomass. Compared to the other overall reactions, the overall reaction corresponding to the known pathway produced the smallest amount of biomass.

**Table 2:** Growth-linked metabolism of 1,2,4-TCB

Overall reaction	Maximum growth yield^a ^(g biomass/mmol 1,2,4-TCB)	1,2,4-TCB uptake^b ^(mmol/gDW/h)
[K]	0.048	4.2
[1]	0.053	4.3
[2]	0.053	4.3
[3]	0.078	5.4
[4]	0.059	4.6
[5]	0.055	4.4
[6]	0.075	5.2
[7]	0.059	4.5

^a ^MFA was used to predict the maximum growth yield with 1,2,4-TCB as the sole carbon source.

^b ^Estimated uptake rate of 1,2,4-TCB when the growth yield was maximized, as predicted by MFA.

**Figure 2:**
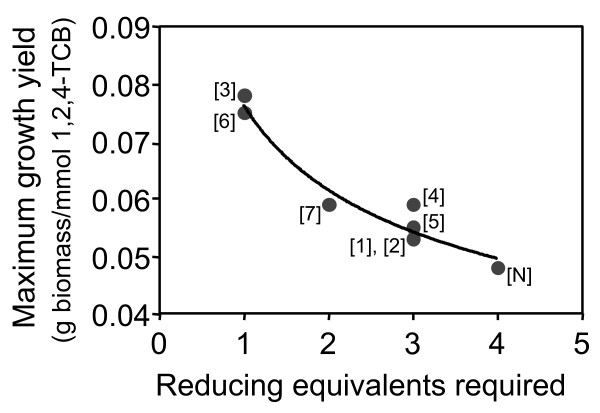
**Relationship between growth yield and reducing equivalents**. Each overall reaction was implemented individually, and MFA was used to predict the maximum growth yield. The number of reducing equivalents is equal to 1/2 the number of electrons required plus the net number of NAD(P)H molecules used to integrate the degradation products into central metabolic pathways. The points are labelled according to the corresponding overall reaction. The best-fit line is drawn to guide the eye.

Although the pathways uptake similar amounts of 1,2,4-TCB, the reducing power required forced the cell to utilize the carbon source differently. Since all of the pathways required reducing power in the form of electrons or NADH, it is important to investigate the cellular processes involved in generating the required reducing power. Pathways requiring electrons diverted some 1,2,4-TCB away from the production of biomass in order to produce acetaldehyde and subsequently the electron donor, acetate (see Methods section). In some cases (overall reactions [K], [2], [5] and [6]), NADH was required to complete the biodegradation of 1,2,4-TCB. Flux analysis showed that the acetaldehyde dehydrogenase reaction (acetaldehyde + CoA + NAD^+ ^→ acetyl-CoA + NADH + H^+^), involved in toluene metabolism, was used to generate NADH within the cell. Figures 1 and S1 show the flux through the electron donating reaction, the flux to NADH from central carbon metabolism and toluene metabolism, and the flux to acetate from either the cysteine or urea metabolism pathways. Additionally, we have characterized the reducing power by counting the reducing equivalents required to degrade 1,2,4-TCB and integrate the biodegradation product(s) into the central metabolic pathways. The number of reducing equivalents for each pathway is equal to half the number of electrons plus the net number of NAD(P)H molecules used. Figure 2 clearly illustrates the trade-off between reducing power and growth yield, demonstrating that novel biodegradation pathways requiring the smallest number of reducing equivalents may be more desirable when maximizing growth.

**Figure 3:**
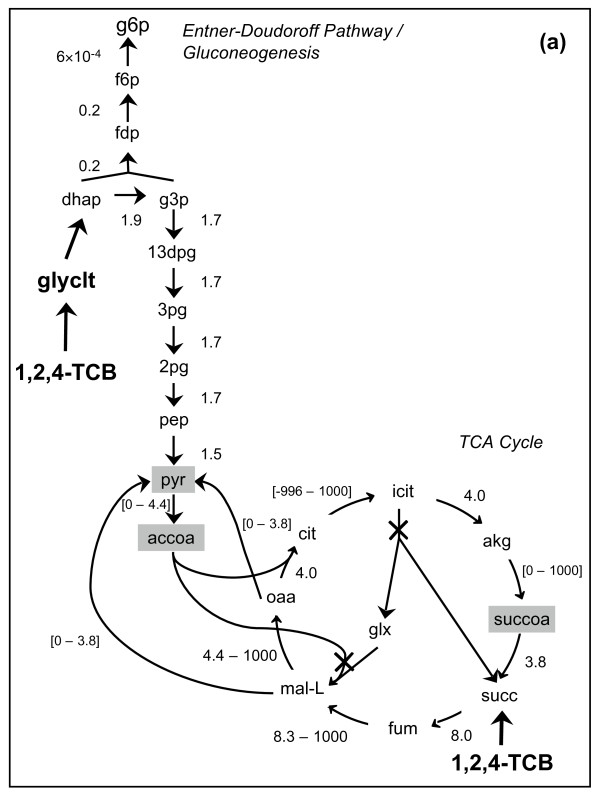
**Flux distribution in central metabolic pathways for growth on 1,2,4-TCB**. Flux ranges for the central metabolic pathways are shown where black indicates essential reactions, square brackets denote substitutable reactions, and blocked reactions are marked with an "X". The overall reactions for eight different pathways were implemented individually; the flux distribution resulting from implementation of overall reaction [K] is given here. Flux distribution maps for all eight overall reactions studied are shown in Additional File 2. The units for the flux values are mmol/gDW/h. A negative flux indicates the reaction can proceed in the reverse direction compared to what is shown. Compound abbreviations are given in the appendix. Shaded boxes indicate biomass precursors.

Classification of the reactions in the central metabolic pathways provided a basis for understanding the cell's metabolic behavior for each overall reaction implemented. Flux variability analysis (FVA) [41] was used to classify reactions as essential (non-zero flux required for optimal biomass production), substitutable (can carry a zero or non-zero flux for optimal biomass production), or blocked (does not carry any flux at optimal biomass production). FVA showed that regardless of which overall reaction was implemented, 98% of the reactions had the same classification. The remaining 2% (19 reactions) represented differences in the cellular physiology leading to different amounts of biomass produced (Table 2). Thirteen of these reactions were involved in integrating the products of TCB biodegradation into the existing metabolism of the cell. Additionally, six reactions were involved in central metabolic pathways (Entner-Doudoroff pathway, gluconeogenesis, and the TCA cycle). Differences in the metabolic behavior of the cell, described in detail below, are summarized using flux distribution maps. As an example, the flux distribution map for the implementation of overall reaction [K] is given in Figure 3 (see Additional File 2 for the flux maps for all overall reactions examined). Note that only a portion of the *P. putida *metabolic network is given to allow for easy comparison of the differences due to implementation of the various biodegradation pathways. Variability in the flux values comes from the substitutable nature of the reactions. For example, in the metabolism of citrate to form isocitrate, the cell can use the aconitase reaction (reaction citrate → isocitrate) or a combination of aconitase half-reaction A citrate hydro-lyase and aconitase half-reaction B isocitrate hydro-lyase (citrate → aconitate + water → isocitrate). Overall, the net flux from citrate to isocitrate is approximately 4 mmol/gDW/h.

One key difference was the utilization of the glyoxylate shunt, which is composed of the isocitrate lyase and the malate synthase reactions. The purpose of the glyoxylate shunt is to synthesize four-carbon compounds, such as oxaloacetate, required for biosynthetic reactions and is primarily utilized during growth on two-carbon substrates [42]. Other differences included utilization of the malic enzyme reaction in order to synthesize pyruvate from malate and employing the pyruvate kinase reaction to form pyruvate from phosphoenolpyruvate and one molecule of ATP.

(i) *Overall reaction [K]*. In this case, glycolate and succinate were the products of biodegradation. The glycolate and glycerophospholipid pathways were utilized to form dihydroxyacetone phosphate (DHAP), which entered the Entner-Doudoroff pathway. Succinate entered the TCA cycle directly. The malic enzyme and oxaloacetate decarboxylase reactions were substitutable, and the malate synthase reaction was blocked. The pyruvate kinase reaction was an essential reaction. The isocitrate lyase, part of the glyoxylate shunt, was substitutable; however, the flux through this reaction was very small (0 - 1 × 10^-8 ^mmol/gDW/h).

(ii) *Overall reaction* [1]. Here, 1,2,4-TCB was degraded to form glycolate and fumarate. Glycolate was first metabolized to form DHAP and then entered the Entner-Doudoroff pathway, while fumarate entered the TCA cycle directly. Additionally, fumarate was used to replenish oxaloacetate levels; therefore the glyoxylate shunt was blocked.

(iii) *Overall reaction* [2]. The flux distribution for implementation of overall reaction [2] was quite similar to that for overall reaction [1]. However, in this case, glycolate and malate were formed. Glycolate followed the same path as previously described, and malate entered the TCA cycle directly. The pyruvate kinase reaction was required, and the glyoxylate shunt was blocked since oxaloacetate could be formed from malate.

(iv) *Overall reactions* [3] *and* [4]. Catechol, the product of reactions [3] and [4], was metabolized via the toluene pathway to form pyruvate and acetaldehyde, key metabolites involved in cellular respiration [42]. Pyruvate was further metabolized to form amino acids, revealing a relatively direct route from the biodegradation products to form biomass precursors. FVA indicated that the malic enzyme and pyruvate kinase reactions were blocked for both overall reactions [3] and [4]. For overall reaction [3], the malate synthase reaction was blocked, whereas for overall reaction [4], the malate synthase reaction was substitutable, with a small range of flux values. Although overall reactions [3] and [4] both formed pyruvate and acetaldehyde, overall reaction [4] required more electrons, thereby reducing the amount of biomass produced. Additionally, overall reaction [3] did not have any oxygen requirements, another reason for the large amount of biomass.

(v) *Overall reactions* [5] *and *[6]. The product of these biodegradation reactions was muconolactone, which was metabolized to form acetyl-CoA and succinate via the β-ketoadipate pathway. Both acetyl-CoA and succinate entered the TCA cycle directly. The isocitrate lyase reaction was required; however, the malic enzyme reaction was substitutable. Overall reactions [5] and [6] both generated direct entry points into the TCA cycle; however, reaction [6] did not require any electrons and only utilized one molecule of oxygen. Therefore, it produced a larger amount of biomass.

(vi) *Overall reaction* [7]. This reaction produced 2-hydroxymuconate, which was metabolized to form acetaldehyde and pyruvate via the toluene pathway. Since this did not provide a direct entry point into the TCA cycle, the glyoxylate shunt was utilized to synthesize oxaloacetate. The isocitrate lyase reaction was essential, while the malate synthase reaction was substitutable.

Utilization of growth media components also influenced biomass production. The organism required certain nutrients in order to meet its energetic demands and optimal utilization of these nutrients impacted biomass production. We used shadow prices to estimate the usefulness of a metabolite in increasing the amount of biomass produced. This analysis indicated that availability of oxygen, phosphate, and sulfate limited biomass production (Table 3). Oxygen availability in particular had the largest influence on biomass production. Although this oxygen limitation could be exaggerated by the observation that the *P. putida *model may be missing reactions that enable more oxygen-efficient metabolism [36], oxygen is known to be a limiting factor in biodegradation [43]. The overall reaction corresponding to the known pathway had the least negative shadow price for oxygen. This means that compared to the other reactions, biomass production using the overall reaction corresponding to the known pathway was least sensitive to increases in oxygen availability.

**Table 3:** Shadow price analysis of selected media components

Overall reaction	**Fe**_2_	H^+^	H_2_O	NH_4_	O_2_	P_i_	SO_4_
[K]	0^a^	0.002	0	0	-0.011	-0.002	-0.004
[1]	0	0.002	0	0	-0.012	-0.002	-0.004
[2]	0	0.002	0	0	-0.012	-0.002	-0.004
[3]	0	0.002	0	0	-0.023	-0.002	-0.005
[4]	0	0.002	0	0	-0.015	-0.002	-0.004
[5]	0	0.002	0	0	-0.013	-0.002	-0.004
[6]	0	0.002	0	0	-0.021	-0.002	-0.005
[7]	0	0.002	0	0	-0.015	-0.002	-0.004

^a ^Shadow price is given as g biomass/mmol of metabolite.

**Figure 4:**
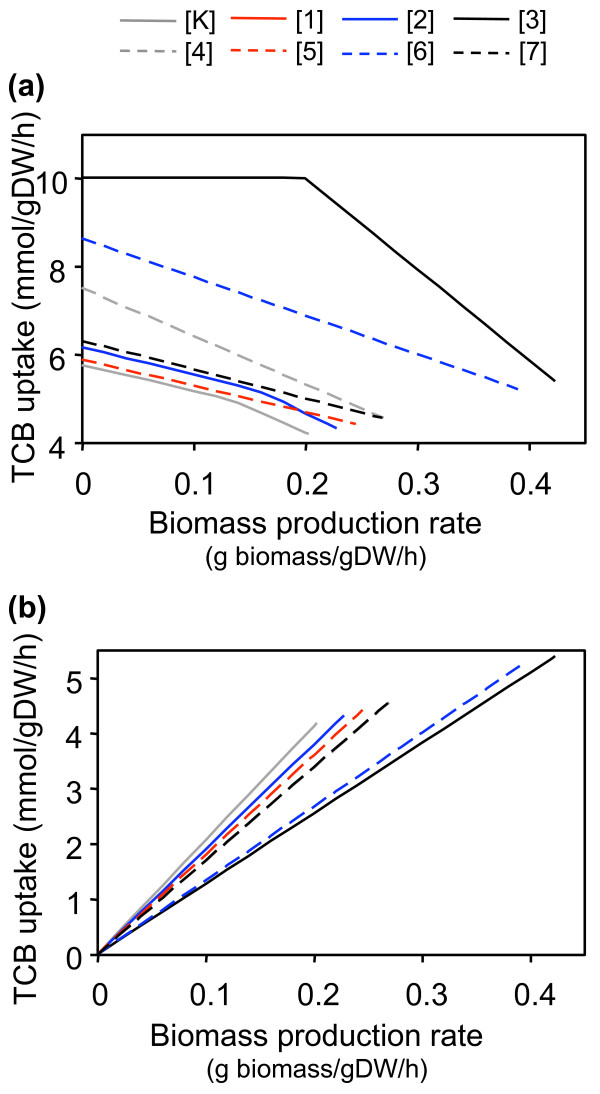
**Relationship between biomass production and metabolism of 1,2,4-TCB**. Each overall reaction was implemented individually, and MFA was used to predict the (a) maximum 1,2,4-TCB uptake rate and (b) minimum 1,2,4-TCB uptake rate required for a given amount of biomass.

There was a direct relationship between maximizing biomass production and maximizing the biodegradation of the xenobiotic compound. To determine the relationship between 1,2,4-TCB uptake and the production of biomass, the biomass production rate (g biomass/g DW/h) was fixed at different values, and MFA was used, where the objective function was to maximize 1,2,4-TCB uptake. These calculations revealed a trade-off between cell growth and biodegradation (Figure 4a).

Implementation of some of the reactions resulted in a smooth monotonic decrease in 1,2,4-TCB uptake with increasing biomass produced. In other cases, the line changed slope, indicating a change in the metabolic behavior of the cell (i.e., a metabolite became limiting). The known pathway was an extreme case, and for a given amount of biomass produced, required the least amount of 1,2,4-TCB. When the objective was to maximize the uptake rate of 1,2,4-TCB (achieve maximal bioremediation), overall reactions [3] and [6] were superior alternatives to the known pathway. Overall reaction [3] allowed the cell to uptake the maximum amount of 1,2,4-TCB without diminishing biomass production until oxygen became limiting, indicated by the change in slope. The same analysis was performed with glucose as the sole carbon source, and in that case, the trade-off between carbon source uptake and biomass production was not observed. The cell was able to uptake the maximum amount of glucose without any effect on biomass production. As described above, 1,2,4-TCB metabolism is largely influenced by reducing requirements, reactions used to integrate the biodegradation product(s) into the existing metabolism, the route to biomass precursors, and oxygen utilization. For these reasons not all of the 1,2,4-TCB taken up by the cell is used to generate biomass. In contrast, no additional reducing power or oxygen is required for glucose metabolism, and the cell can fully harness the potential of this carbon source for cell maintenance, and ultimately to generate biomass. Together, these results indicate that when utilizing 1,2,4-TCB as the sole carbon source, xenobiotic metabolism and cell growth were competing objectives.

The novel pathways generated by BNICE utilized the carbon source for the production of biomass more efficiently than the known pathway. We found that for implementation of all eight overall reactions studied, when the cell was not producing the maximum possible biomass, 1,2,4-TCB was not fully metabolized, and one or more carbon compounds were excreted other than bicarbonate and carbon dioxide, which are common by-products of cell growth. For overall reactions [K], [1], [2], and [7], glycolate was excreted to the extracellular compartment, for overall reactions [3] and [4], both catechol and glycolate were excreted, and glycolate and 3-oxoadipate were excreted in the cases of overall reactions [5] and [6]. Therefore, we performed another study to investigate the efficient utilization of the carbon source for the production of biomass. Here, biomass production was fixed at different values, and MFA was applied to estimate the minimum 1,2,4-TCB uptake rate required to obtain the given biomass production rate. As expected, increasing amounts of 1,2,4-TCB were required to generate more biomass (Figure 4b). Although overall reactions [K] and [3] were still the extreme cases, the results were opposite of those shown in Figure 4a; overall reaction [3] required the least amount of 1,2,4-TCB for a given amount of biomass, representing the most efficient case, while overall reaction [K] represented the least efficient option. Additionally, the ordering of the lines follows the trend observed in Table 2. This further demonstrates that the pathways corresponding to overall reaction [3] are attractive alternatives to the known biodegradation route of 1,2,4-TCB.

The cell was able to distinguish between alternative biodegradation routes and select the reaction or set of reactions that maximized biomass production. We implemented all eight reactions simultaneously, allowing all of the reactions to carry a flux, and applied MFA to predict the maximum biomass produced. The cell preferred to utilize overall reaction [3] alone or in combination with overall reaction [6] in order to maximize biomass production. We applied reduced cost analysis to estimate the cost associated with utilizing each overall reaction. We have used this analysis to understand how each overall reaction affects the cellular metabolism when the cell is given the option to use all of the reactions. Reduced cost analysis indicated that if any of the other reactions were forced to carry a flux, the maximum biomass produced would decrease (Table 4). This result agrees with those obtained from implementing the reactions individually, where overall reactions [3] and [6] provided the largest amount of biomass (Table 2). These reactions utilized a more direct route to form biomass precursors and had the lowest requirement of oxygen and reducing power (Figure 2). In addition, if the flux through overall reaction [3] or [6] was increased above the optimal range, less biomass would be produced, again demonstrating the trade-off between 1,2,4-TCB uptake and biomass production.

**Table 4:** Reduced cost analysis

Overall reaction	Range of flux values^a^	Reduced cost^b^
[K]	0	-0.045
[1]	0	-0.036
[2]	0	-0.036
[3]	4.7 - 5.4	-0.016
[4]	0	-0.034
[5]	0	-0.034
[6]	0 - 0.7	-0.016
[7]	0	-0.034

^a ^In the formulation of the linear optimization problem, all eight overall reactions were allowed to carry a flux, and MFA predicted the maximum growth yield (0.078 g biomass/mmol 1,2,4-TCB). FVA was used to determine the range of flux values possible in order to maximize biomass production.

^b ^Reduced cost is given as g biomass/mmol 1,2,4-TCB.

### Determination of physiologically feasible flux profiles

Since implementation of any pathway with the same overall reaction will produce the same flux distribution, we utilized thermodynamic properties of the reactions, estimated using a group contribution method [23], to study the effect of individual pathways on the metabolism of *P. putida*. Here, we studied pathways corresponding to the best overall reactions based on MFA ([3], [4], and [6]). For comparison, we also studied novel pathways corresponding to the known overall reaction. We examined pathways that involved only known intermediates (i.e., catalogued in the Chemical Abstracts Service (CAS) Registry or KEGG database) and were shorter than or equal to the length of the known pathway. We selected these screens based on the assumption that pathways involving known intermediates will require a lesser amount of genetic engineering in order to implement them into a host organism, since enzymes evolve to degrade compounds that are structurally similar to their natural substrate. These screening criteria resulted in a total of 1,195 novel pathways.

In order to identify thermodynamically feasible biodegradation pathways, we first identified pathway reactions that were able to carry a flux. FVA, combined with thermodynamic constraints, was applied to classify reactions as essential, substitutable, or blocked. Pathways that included any blocked reactions were not examined further. Thermodynamics-based metabolic flux analysis (TMFA) was then utilized to predict flux profiles, coupling thermodynamic and mass balance constraints. In each of the four cases studied, the distinct pathway reactions were implemented into the metabolic model of *P. putida*. For example, there were 500 pathways that corresponded to overall reaction [3]; however, only 67 distinct reactions were used in many different combinations to form the 500 pathways. Only the distinct reactions were incorporated into the model and used in FVA and TMFA, since this subset accounted for all possible permutations.

Thermodynamic properties of the reactions comprising the pathway led to physiological differences in cellular metabolism. In all four cases, the thermodynamic constraints affected the maximum biomass produced, where less biomass was predicted compared to that obtained when only mass balance constraints were utilized. FVA revealed that 10 reactions that were blocked with only mass balance constraints were classified as essential reactions when including thermodynamic constraints. Additionally, some essential and substitutable reactions became blocked. Therefore, the addition of thermodynamic constraints caused carbon flux to be redirected through alternative reactions that resulted in lower biomass production.

Incorporation of thermodynamic properties of the reactions identified 813 pathways out of the 1,195 possible novel pathways that allowed the cell to utilize 1,2,4-TCB as the sole carbon source, produced some amount of biomass, and were free of thermodynamic infeasibilities. When the distinct pathway reactions corresponding to overall reactions [K], [3], and [6] were implemented, all of the biodegradation reactions were essential or substitutable, indicating that all were able to carry a flux and produce physiologically feasible flux profiles (Table 5). Although some of the reactions had a positive standard millimolar free energy estimate, there was a range of metabolite activity that allowed these reactions to proceed in the forward direction. For example, there were 319 pathways (64%) corresponding to overall reaction [3] that involved a large uphill step where Δ_*r*_*G*'^m ^was 12.5 ± 5.4 kcal/mol (three standard errors). These pathways were still able to produce the same amount of biomass as pathways without this step. Therefore, solely studying the free energy of individual reactions is not enough to determine the feasibility; rather, it is necessary to investigate reactions within the context of the entire biodegradation pathway [44] and consider other metabolic reactions taking place within the cell. In the case of pathways corresponding to overall reaction [4], five of the distinct pathway reactions were classified as blocked. Using these results to screen the pathways, we identified 297 pathways corresponding to overall reaction [4] that resulted in physiologically feasible flux profiles (Table 5).

**Table 5:** Evaluation of novel pathways with thermodynamic constraints

		Number of pathways		
		
Overall reaction	Maximum growth yield^a ^(g biomass/mmol 1,2,4-TCB)	Total^b^	Met pathway screen^c^	Met TMFA screen^d^
[K]	0.039	1,368	8	8
[3]	0.070	94,558	500	500
[4]	0.041	97,980	679	297
[6]	0.072	26,252	8	8
		
*Total:*		*220,158*	*1195*	*813*

^a ^MFA predicted the maximum growth yield with 1,2,4-TCB as the sole carbon source.

^b ^Pathways obtained using BNICE and pathway search algorithm, up to length 15.

^c ^Pathways that: (i) did not involve any novel intermediates and (ii) were shorter than or equal to the length of the known pathway, which involved 10 steps.

^d ^Pathways that produced physiologically feasible flux profiles (did not involve any blocked reactions).

## Discussion

We have generated novel biodegradation pathways describing the metabolism of 1,2,4-TCB to compounds with known metabolism. We estimated the cell's ability to utilize these novel pathways for growth with 1,2,4-TCB as the sole carbon source. We have shown that the cell is able to achieve bioremediation by coupling 1,2,4-TCB utilization and biomass production. Using mass balance and thermodynamic constraints, we identified physiologically feasible biodegradation pathways that are attractive alternatives to the known pathway. To our knowledge, this is the first study of the effect of engineering a novel biodegradation pathway into a host organism by estimating how the pathway influences the organism's existing metabolism.

This work may provide some explanation about the selective pressure for the known pathway. The complete route from 1,2,4-TCB to chloroacetate and succinate is shown to occur in *Pseudomonas *sp. strain PS12 [40]. Based on the shadow price analysis (Table 3), one could argue that this pathway evolved because it is able to more efficiently utilize the available oxygen. Although it is not clear what additional physiologic constraints the environment imposes and how those constraints influence biodegradation pathways observed in nature, this might lend some evidence as to why the known pathway has been elucidated.

This study focused on the utilization of 1,2,4-TCB as the sole carbon source and estimated the impact of novel biodegradation pathways on the physiology of the cell. We did not account for the availability and utilization of multiple substrates for growth and biomass production, which is the case in many environmental systems. Additionally, we have assumed that *P. putida *is the only organism utilizing 1,2,4-TCB. However, consortia of microorganisms may also be used to metabolize xenobiotics sequentially or in concert, and it is important to consider biodegradation of multiple compounds via several different organisms [45]. Future work may be aimed at addressing these issues in order to more fully understand the fate of compounds in the environment and the cellular feasibility of novel biodegradation pathways.

## Conclusions

The BNICE prediction tool generated hundreds of thousands of novel pathways to degrade 1,2,4-TCB to compounds with known metabolism, and subsequent screening identified a subset of pathways that may be engineered into a host organism. Based on this work, we propose that the pathways corresponding to overall reaction [3] that met the pathway and TMFA screens are the most attractive targets for metabolic engineering of *P. putida *to degrade 1,2,4-TCB. BNICE predicted 500 pathways that met the pathway and TMFA screens, utilizing three 3^rd^-level EC numbers: EC 1.3.1 (forward and reverse), EC 1.97.1, and EC 3.8.1. These pathways, listed in Additional File 3, offer the largest growth yield and biodegradation potential (i.e., largest uptake of the xenobiotic), making them ideal routes to employ in the degradation of 1,2,4-TCB.

## Methods

### Generation of novel biodegradation pathways

We have developed a computational framework, called BNICE, for the prediction of novel biodegradation pathways for anthropogenic compounds [22]. BNICE utilizes a systematic formulation of enzyme reaction rules describing the transformation of biochemical compounds. The rules are based on the EC classification system, where each enzyme is assigned a four-digit number: EC i.j.k.l. The first level, *i*, designates the type of chemistry performed by the enzyme; the second level, *j*, designates the functional group undergoing the transformation; the third level, *k*, designates the cofactors required for the reaction; and the fourth level, *l*, designates the reactants participating in the reaction. Upon examination of the enzyme reactions catalogued in the Kyoto Encyclopedia of Genes and Genomes (KEGG) database [39], it was discovered that the first three levels of classification describe the overall action of an enzyme and can be used to define generalized enzyme reactions, termed enzyme reaction rules. BNICE iteratively applies the reaction rules in order to generate novel biochemical compounds and reactions. First, each starting compound is evaluated to determine if it contains the functional group required to undergo the reaction. The reaction rules are then applied and all possible products are generated. In the next iteration, the reaction rules are applied to the products from the previous generation, and this application of the rules is repeated in successive generations until no new compounds are created or the maximum number of iterations has been reached.

We have compiled a set of enzyme reaction rules that provides broad coverage of known biodegradation reactions and predicts novel reactions. This set includes reaction rules developed based on curation of the KEGG database and the *i*JR904 genome-scale model of *E. coli *metabolism [19-21,46] and reaction rules relevant to biodegradation [22]. The complete set of reaction rules is able to effectively navigate the chemistry of biodegradation, capturing existing reactions, adding new connections between known compounds, and generating novel reactions.

We employed BNICE to generate novel biodegradation pathways for 1,2,4-trichlorobenzene. We determined the enzyme reaction rules necessary to reproduce the known pathway catalogued in the UM-BBD and implemented those rules into the BNICE framework in order to generate novel routes to compounds with known intermediary metabolism. Ten reaction rules were required to reproduce the known pathway and included oxidoreductase, hydrolase, and isomerase enzymes [22]. The reaction network generated by BNICE, starting from 1,2,4-TCB, served as the basis for the present study.

### Metabolic flux analysis of *P. putida *metabolism

Metabolic flux analysis (MFA) is used to estimate the distribution of fluxes through the reactions in a metabolic network while satisfying the mass, energy, and redox requirements of the cell. This analysis is based upon the assumption that the net production of intracellular metabolites is zero. This quasi-steady state assumption is given by

where **N **is the *m *× *n *matrix containing the stoichiometric coefficients for the *m *metabolites in each of the *n *reactions of the metabolic network and *v *is an *n *× 1 vector of fluxes through the *n *reactions. Eq. 1 establishes a set of linear constraints on the cell. However, in metabolic networks, the system is underdetermined, and the number of reactions typically exceeds the number of metabolites; thus, there is a set of solutions [47]. A particular solution can be found using linear optimization, subject to an objective function. This function may be to maximize cell growth or yield of a particular metabolite. MFA has many different applications in biotechnology. For example, it has been used to investigate the maximum growth rate of a cell [48,49], to predict metabolic behavior of the cell following adaptive evolution [50], to predict yield of biochemicals [51], to examine the redundancy in metabolic networks and account for cell regulation [52], and to predict gene pairs that exhibit compensatory rescue of cell growth [53].

In this work, the MFA studies were performed using the *i*JN746 model for *Pseudomonas putida *metabolism [36]. The model contained 950 reactions and 911 metabolites located in three different compartments: extracellular space, cytoplasm, and periplasm. Aerobic growth was modeled using the *in silico *M9 minimal medium [36] under a specific set of constraints on the metabolites that could be taken up by the cell or excreted to the extracellular compartment (described below). The model was augmented by adding biodegradation reactions and pathways obtained using the BNICE framework, with 1,2,4-TCB as the sole carbon source. Linear programming was used to solve the optimization problem where we examined three objective functions: maximum biomass production (a measure of cell growth), maximum uptake of 1,2,4-TCB (an indication of the rate of biodegradation), and minimum utilization of 1,2,4-TCB.

#### *In silico *growth constraints

Constraints on the intracellular fluxes and exchange reactions were applied in the same manner as previously done by others [47,54]. The flux through intracellular fluxes was constrained to ν_min _≥ -1000 mmol/gDW/h and ν_max _≤ 1000 mmol/gDW/h and ν_min _≥ 0 mmol/gDW/h and ν_max _≤ 1000 mmol/gDW/h for reversible and irreversible reactions, respectively. The cell was allowed to take up the following metabolites given by the M9 minimal medium: CO_2_, Co_2 _^+ ^Fe_2 _^+^, H^+^, H_2_O, Na_2 _^+^, Ni_2 _^+^, NH_4_, P_*i*_, and SO_4_. The flux through the exchange reactions for these media components was constrained to ν_min _≥ -100 mmol/gDW/h and ν_max _≤ 1000 mmol/gDW/h, where a negative flux indicates the uptake rate of the metabolite. The oxygen uptake rate was constrained to ν_min _≥ -18.5 mmol/gDW/h [36], and the uptake rate for 1,2,4-TCB was constrained to ν_min _≥ -10 mmol/gDW/h. All other external metabolites, with the exception of those incorporated into biomass, were only permitted to be excreted from the cell, and their exchange fluxes were constrained to ν_min _≥ 0 mmol/gDW/h and ν_max _≤ 1000 mmol/gDW/h. The external metabolites are listed in Additional File 4.

#### Shadow price and reduced cost

Given the optimal flux distribution obtained from MFA, one can study the sensitivity of the objective function to metabolites and reactions present in the model using the shadow price and reduced cost, respectively [55]. The shadow price indicates the rate at which the objective function increases when the supply of a particular metabolite is increased. A positive shadow price means the metabolite is in excess and can be secreted from the cell. A shadow price of zero means the metabolite cannot be used to increase growth. A negative shadow price indicates a limiting metabolite; increasing its availability will increase biomass production. The reduced cost is the amount by which the objective function would increase when the flux through a reaction is increased by a single unit. Shadow price and reduced cost analysis were used to characterize the optimal solution and infer the effect of cellular resources and metabolic reactions.

### Estimation of thermodynamic properties of biochemical reactions

The Gibbs free energy of reaction provides a means of assessing the thermodynamic properties of reactions. Group contribution has been shown to be a valuable to tool to provide a priori estimates of the energetic feasibility of biodegradation reactions [44]. Therefore, we employed a group contribution method [23] to estimate the reaction energy of the biodegradation reactions generated in the BNICE framework. The standard Gibbs free energy of reaction with a 1 mM reference state, Δ_*r*_*G*'^*m*^, was estimated for aqueous solution at a temperature of 298 K. The 1 mM reference state was used for all reactants, with the exception of H^+^, H_2_O, H_2 _and O_2_. H^+ ^and H_2_O are already incorporated into the free energy estimate. For H_2 _and O_2_, the saturation concentration was used as the reference state, 3.4 × 10^-5 ^M and 5.5 × 10^-5 ^M, respectively. Δ_*r*_*G*'^*m *^also included the transmembrane potential and proton gradient for reactions involving transport across the cytoplasmic membrane, where the cytoplasmic and periplasmic pH were 7.2 and 7.7, respectively. Special considerations were incorporated for oxygenase and reductive dechlorination reactions. When estimating the free energy of oxygenase reactions, since much of the free energy release in these reactions is not coupled to the generation of electron carriers, we only utilized the free energy available for cell mass maintenance and growth. One of the reactions generated in the BNICE framework is the reductive dechlorination reaction, which requires two electrons per reaction; however, the source of these electrons is not explicitly known. An ideal electron donor is one whose degree of reductance and Gibbs free energy of dissipation are close to the regularity values observed by Minkevich and Eroshin [56]. Based on these criteria, acetate was deemed to be the best candidate; however, this same analysis can be performed for any electron donor of interest. Acetate is native to *P. putida *metabolism, where it is generated from acetaldehyde.

Thermodynamics-based metabolic flux analysis (TMFA) augments the mass balance constraints of MFA with thermodynamic constraints in order to generate physiologically feasible flux distributions and predict the range of feasible metabolite activities [24]. Since the intracellular metabolite concentration can take on a range of values, we estimated the actual free energy change of a reaction, Δ_*r*_*G*', which can differ from Δ_*r*_*G*'^*m*^. Δ_*r*_*G*' accounted for a minimum and maximum metabolite activity of 10^-5 ^M and 0.02 M, respectively, and was allowed to vary within three standard errors. The thermodynamic constraints prevent a reaction from carrying a positive flux if Δ_*r*_*G*' is positive, thereby requiring the flux distribution and metabolite activity profiles to follow the second law of thermodynamics. Special thermodynamic constraints were applied to reactions for which the free energy could not be calculated such that the resulting flux distribution was consistent with thermodynamic analysis.

The concentrations of the extracellular components used as growth nutrients were fixed to concentrations found in the M9 media [57] and based on the bounds set by Henry and coworkers [24]. The intracellular species concentration was allowed to vary between 10^-5 ^M and 0.02 M, typical values observed in the cell [58], with the following exceptions. The concentration of H^+ ^was fixed to maintain a pH of 7.2. The lower bound for the concentration of H_2 _and O_2 _was 10^-8 ^M, while the upper bound was their saturation concentration, 3.4 × 10^-5 ^M and 5.5 × 10^-5 ^M, respectively. In addition, the concentration of CO_2 _varied between 0.0001 M and the saturation concentration 0.0014 M. The first step in the pathways corresponding to the known overall reaction had a standard millimolar free energy estimate of 10.6 kcal/mol. In order to provide the necessary driving force for the reaction, the bounds on the concentration of the non-cofactor reactants (1,2,4-TCB and 3,4,6-trichloro-*cis*-1,2-dihydroxycylohexa-3,5-diene) were expanded to 8 × 10^-7 ^M - 0.025 M (compared to the original bounds of 10^-5 ^M - 0.02 M). We then used these expanded bounds in all TMFA calculations for all of the reactions studied.

## Abbreviations

1,2,4-TCB: 1,2,4-trichlorobenzene; 13dgp: 3-phospho-D-glyceroyl phosphated; 2 h3oppan: 2-hydroxy-3-oxopropanoate; 2 hmc: 2-hydroxymuconate; 2 hmcnsad: 2-hydroxymuconate semialdehyde; 2pg: D-glycerate2-phosphate; 3pg: 3-phospho-D-glycerate; 5odhf2a: 5-oxo-4,5-dihydrofuran-2-acetate; 3oxoadp: 3-oxoadipate; 4 h2opntn: 4-hydroxy-2-oxopentanoate; ac: acetate; acald, acetaldehyde; accoa: acetyl-CoA; akg: 2-oxoglutarate; ccmuac: *cis, cis*-muconate; cit: citrate; dhap: dihydroxyacetone phosphate; f6p: D-fructose 6-phosphate; fdp: D-fructose 1,6-bisphosphate; fum: fumarate; g3p: glyceraldehyde 3-phosphate; g6p: D-glucose 6-phosphate; glyc: glycerol; glyc-R: (R)-glycerate; glyc3p: glycerol 3-phosphate; glyald: D-glyceraldehyde; glyclt: glycolate; glx: glyoxylate; icit: isocitrate; mal-L: L-malate; mucl: muconolactone; oaa: oxaloacetate; op3en: oxopent-4-enoate; oxadpcoa: oxoadipyl-CoA; oxalc: 4-oxalocrotonate; pep: phosphoenolpyruvate; pyr: pyruvate; succ: succinate; succoa: succinyl-CoA.

## Authors' contributions

SDF, LJB, and VH conceived the study. SDF performed the study and wrote the manuscript. All authors read and approved the final manuscript.

## Supplementary Material

Additional file 1 ***P. putida *reactions for growth on 1,2,4-TCB**. The reaction network used to integrate the degradation products of 1,2,4-TCB into *P. putida *metabolism and the cellular processes involved in generating the required reducing power (electrons and/or NADH). Central carbon metabolism includes the Entner-Doudoroff pathway, gluconeogenesis, and the TCA cycle. The acetaldehyde dehydrogenase (acetylating) reaction, involved in toluene metabolism, was also used to generate NADH. The overall reaction for eight different pathways were implemented individually: (a) overall reaction [K]; (b) overall reaction [1]; overall reaction [2]; (d) overall reaction [3]; (e) overall reaction [4]; (f) overall reaction [5]; (g) overall reaction [6]; (h) overall reaction [7]. The units for the flux values are mmol/gDW/h. Compound abbreviations are given in the appendix. Shaded boxes indicate biomass precursors. Click here for file

Additional file 2 **Flux distribution in central metabolic pathways for growth on 1,2,4-TCB**. Flux ranges for the central metabolic pathways are shown where black indicates essential reactions, gray denotes substitutable reactions, and blocked reactions are marked with an "X". The overall reactions for eight different pathways were implemented individually: (a) overall reaction [K]; (b) overall reaction [1]; overall reaction [2]; (d) overall reaction [3]; (e) overall reaction [4]; (f) overall reaction [5]; (g) overall reaction [6]; (h) overall reaction [7]. The units for the flux values are mmol/gDW/h. A negative flux indicates the reaction can proceed in the reverse direction compared to what is shown. Compound abbreviations are given in the appendix. Shaded boxes indicate biomass precursors. Click here for file

Additional file 3 **Novel biodegradation pathways for 1,2,4-TCB**. Excel spreadsheet containing pathways corresponding to overall reaction [3]. These pathways are shorter than or equal to the length of the known pathway, only include known intermediates, and produce thermodynamically feasible flux profiles. Click here for file

Additional file 4 **Exchange metabolites used in the analysis of *P. putida *metabolism**. Excel spreadsheet containing external metabolites included in the model of *P. putida *metabolism. Click here for file
