# Supplementary figures and images for: In silico feasibility of novel biodegradation pathways for 1,2,4-trichlorobenzene
Source: BMC Syst Biol. 2010 Feb 2;4:7. doi: 10.1186/1752-0509-4-7 (PMC2830930; doi:10.1186/1752-0509-4-7)

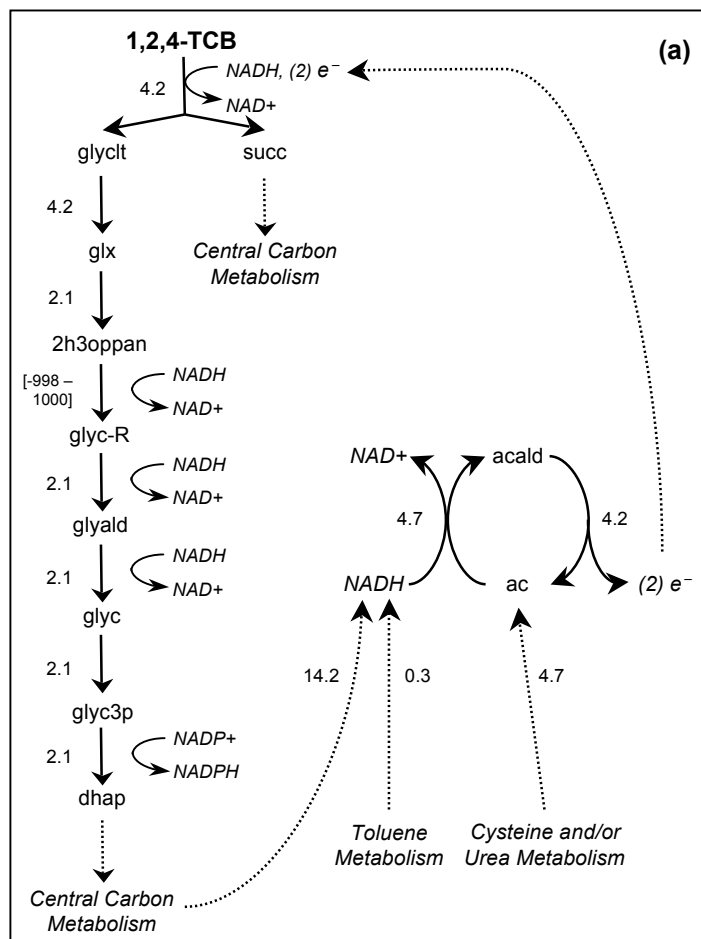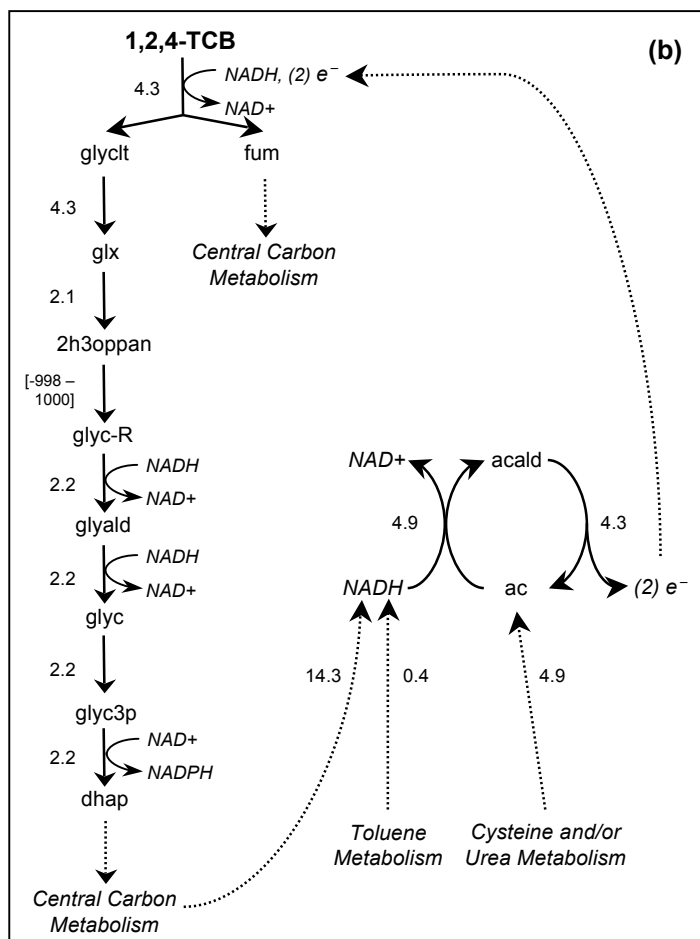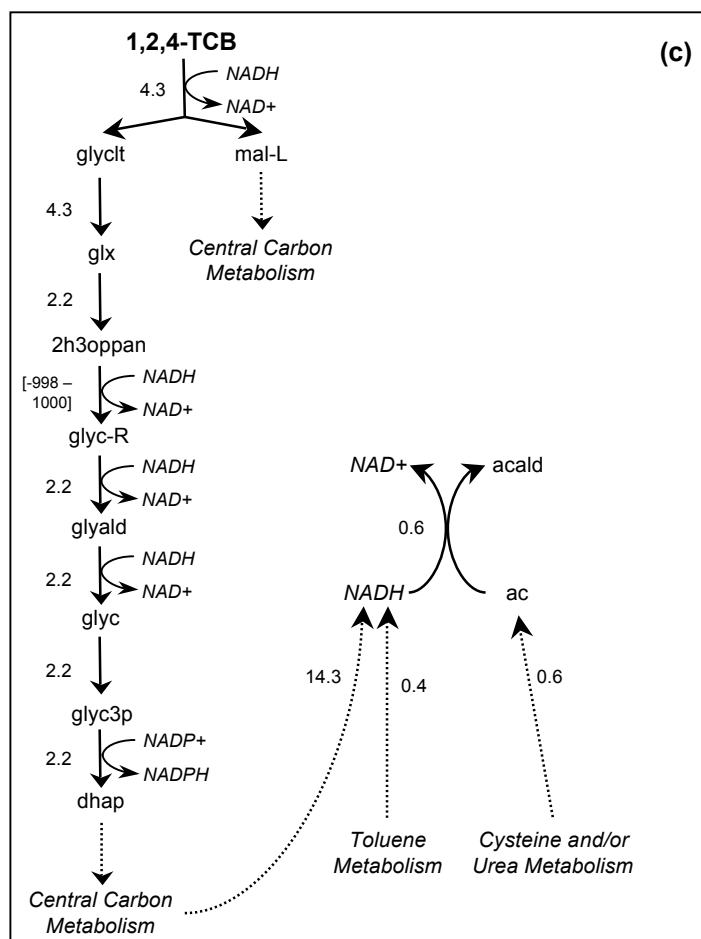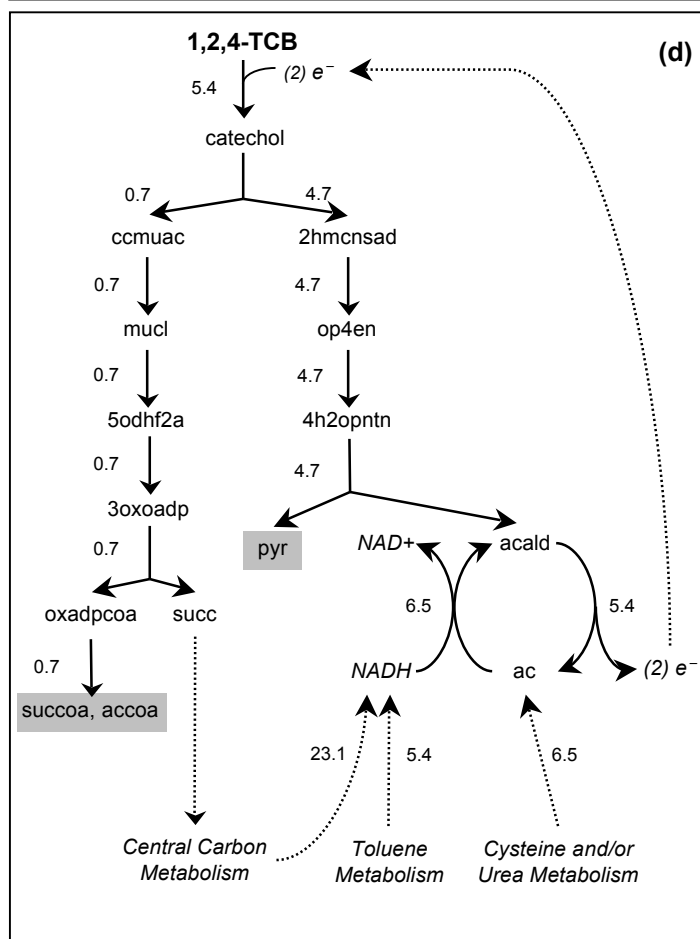

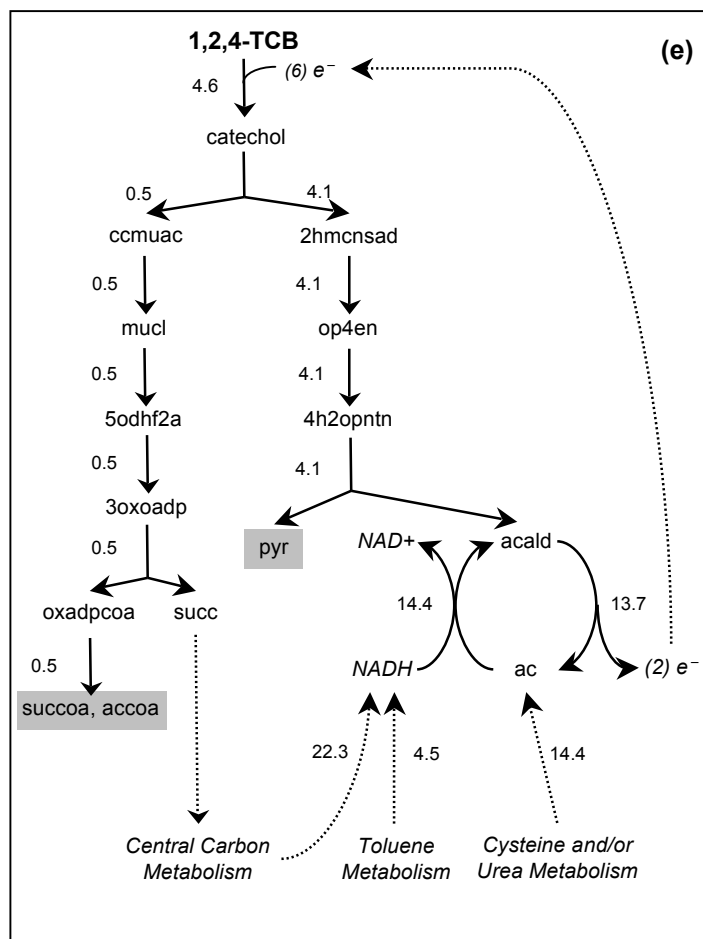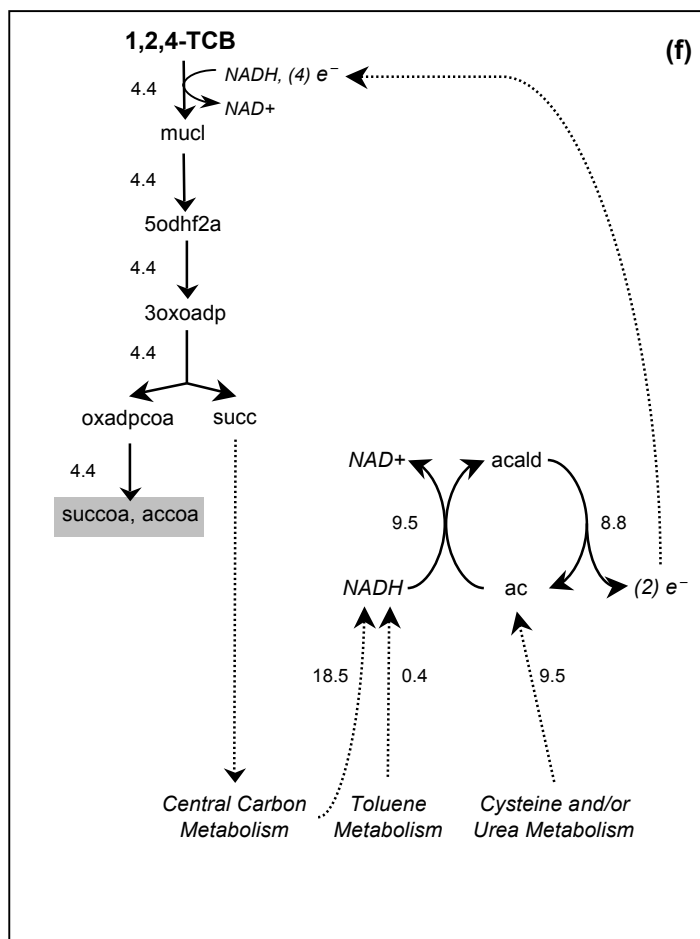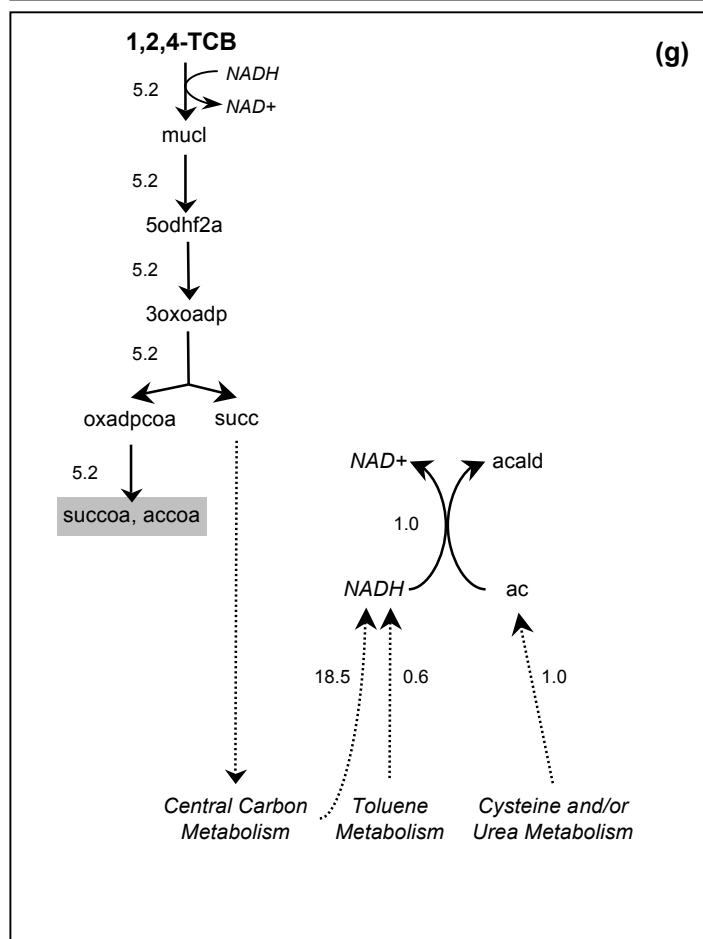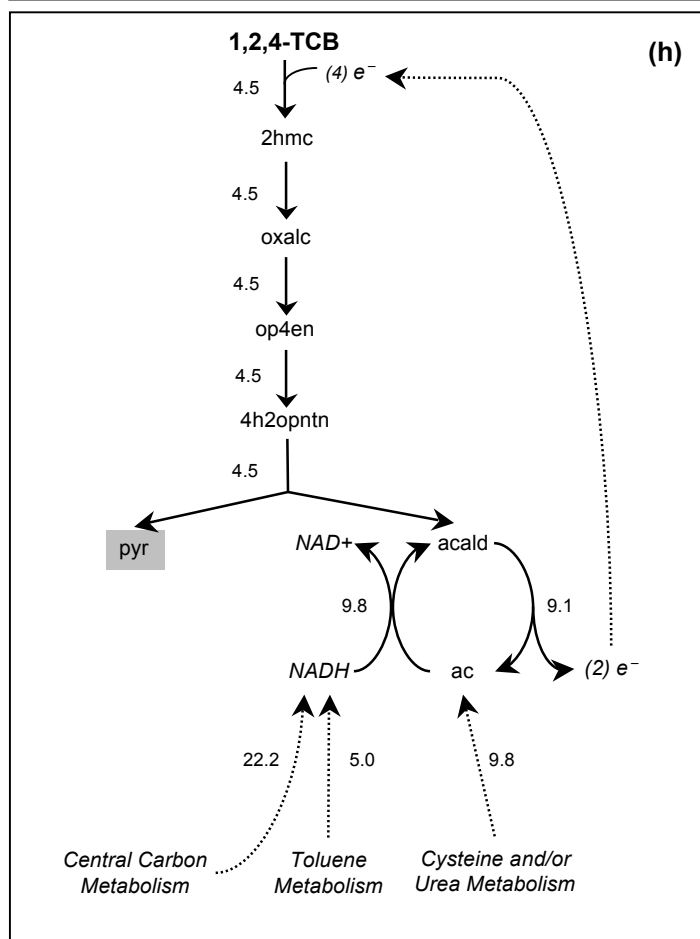

Supplement: Additional file 1 — P. putida reactions for growth on 1,2,4-TCB. The reaction network used to integrate the degradation products of 1,2,4-TCB into P. putida metabolism and the cellular processes involved in generating the required reducing power (electrons and/or NADH). Central carbon metabolism includes the Entner-Doudoroff pathway, gluconeogenesis, and the TCA cycle. The acetaldehyde dehydrogenase (acetylating) reaction, involved in toluene metabolism, was also used to generate NADH. The overall reaction for eight different pathways were implemented individually: (a) overall reaction [K]; (b) overall reaction [1]; overall reaction [2]; (d) overall reaction [3]; (e) overall reaction [4]; (f) overall reaction [5]; (g) overall reaction [6]; (h) overall reaction [7]. The units for the flux values are mmol/gDW/h. Compound abbreviations are given in the appendix. Shaded boxes indicate biomass precursors. [file 1752-0509-4-7-S1.PDF]

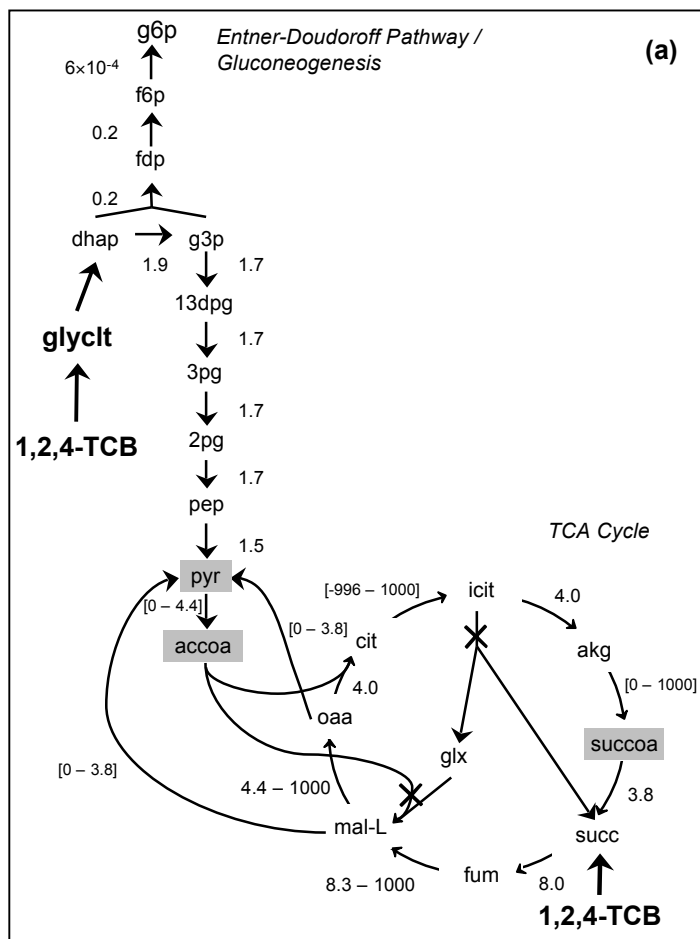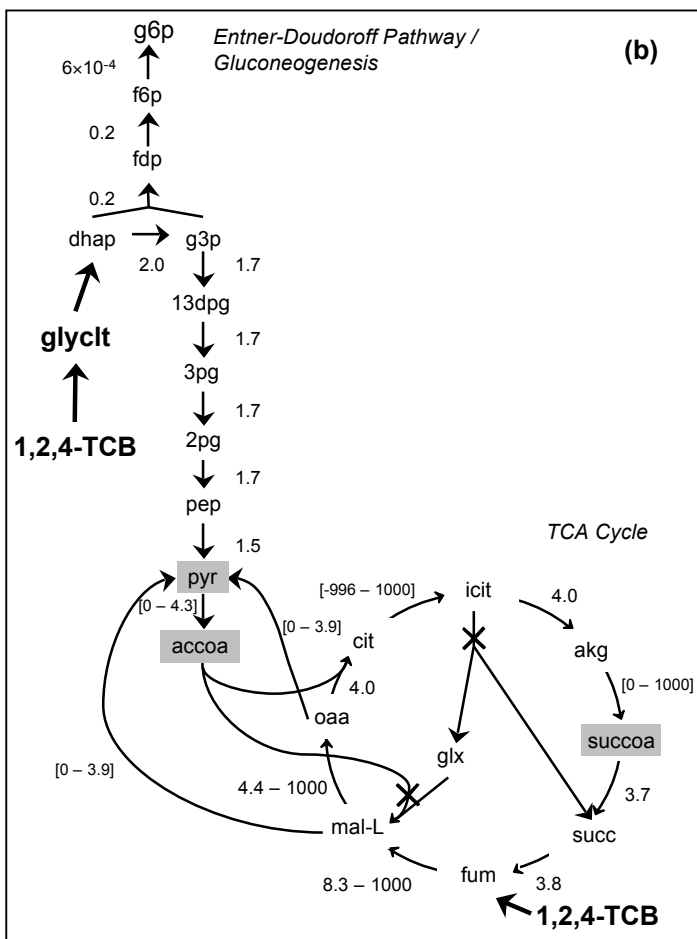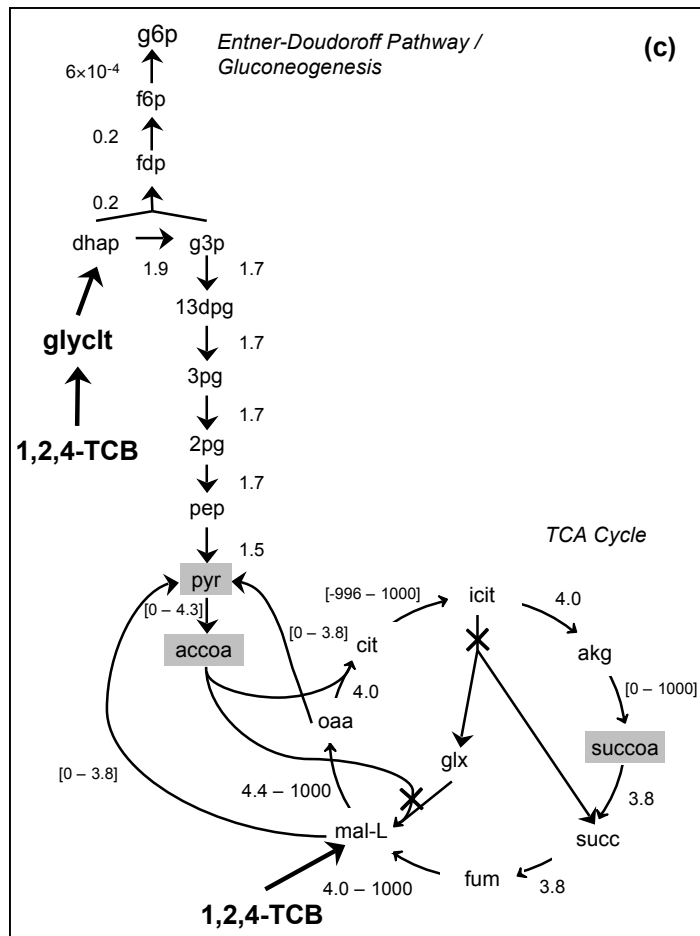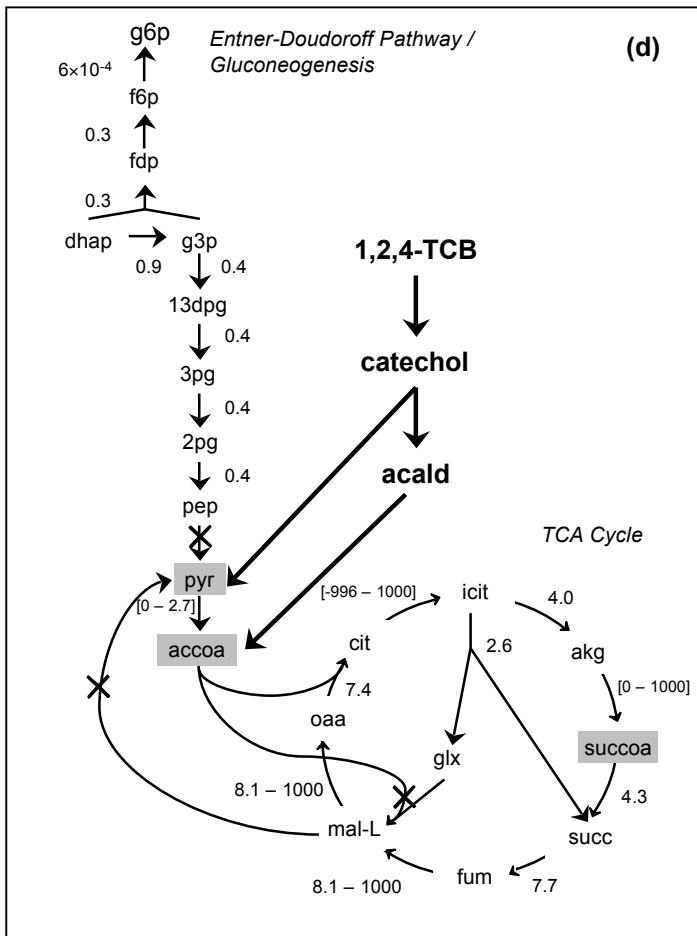

Supplement: Additional file 2 — Flux distribution in central metabolic pathways for growth on 1,2,4-TCB. Flux ranges for the central metabolic pathways are shown where black indicates essential reactions, gray denotes substitutable reactions, and blocked reactions are marked with an "X". The overall reactions for eight different pathways were implemented individually: (a) overall reaction [K]; (b) overall reaction [1]; overall reaction [2]; (d) overall reaction [3]; (e) overall reaction [4]; (f) overall reaction [5]; (g) overall reaction [6]; (h) overall reaction [7]. The units for the flux values are mmol/gDW/h. A negative flux indicates the reaction can proceed in the reverse direction compared to what is shown. Compound abbreviations are given in the appendix. Shaded boxes indicate biomass precursors. [file 1752-0509-4-7-S2.PDF]
